# Effects of moderate static magnetic fields on voltage‐gated potassium ion channels in sympathetic neuron‐like PC12 cells

**DOI:** 10.14814/phy2.70236

**Published:** 2025-03-22

**Authors:** Eri Kaneda, Takafumi Kawai, Yasushi Okamura, Shigeru Miyagawa

**Affiliations:** ^1^ Graduate School of Medicine Osaka University Suita, Osaka Japan; ^2^ Graduate School of Frontier Bioscience Osaka University Suita, Osaka Japan

**Keywords:** neuromodulation, patch clamp, static magnetic fields, sympathetic neuron

## Abstract

While exposure of moderate static magnetic fields (SMF) can alter neuronal excitability, the effects on sympathetic neurons remain underexplored. This study investigates the effects of moderate SMF on Kv channels in the plasma membrane of sympathetic neuron‐like PC12 cells. The current density of Kv channels was significantly lower in the 18‐h magnet‐exposed group, with effects persisting even after the magnet was removed before patch‐clamp measurements. The current density of outward current in the presence of TEA was not different between the two groups, indicating that magnetic field affects TEA‐sensitive Kv channels. To further explore these changes, RNA sequencing was performed on samples from both the Sham and 18‐h magnet‐exposed groups, identifying 37 moderate SMF‐sensitive genes. Changes in mRNA expression levels and KEGG analysis suggested that pathways involved in the inhibition of neuronal excitability, such as GABAB receptor activation and Kir3 channel opening, may be more likely to be activated. In conclusion, moderate SMF is strongly associated with reduced current density in PC12 cells, particularly affecting Kv channels. The present study provides fundamental information on the influence of long‐term SMF exposure on the excitability of sympathetic neurons.

## Graphical abstract

This study investigates the impact of moderate SMF on Kv channels in the sympathetic‐like plasma membrane of PC12 cells. In conclusion, moderate SMF is strongly associated with reduced current density in PC12 cells, particularly affecting Kv channels. The present study provides fundamental information on the influence of long‐term SMF exposure on the excitability of sympathetic neurons.

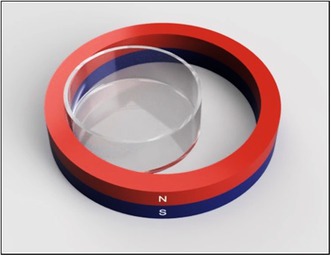

## 1 INTRODUCTION

In the management of hypertension, a certain proportion of patients experience difficulties in achieving adequate blood pressure control. Although the specific definition is beyond the scope of this discussion, the 2017 AHA/ACC Hypertension Guidelines define resistant hypertension as high blood pressure that remains uncontrolled despite the use of three antihypertensive agents, including one diuretic. It is distinguished from more severe cases of refractory hypertension. The causes of resistant hypertension are multifactorial, but one prominent factor is the increased sympathetic nervous activity observed in these patients compared to those who can be controlled hypertension (Grassi et al., 2014), making the assessment and regulation of sympathetic tone a critical challenge.

The mechanism by which sympathetic activation contributes to hypertension includes the release of norepinephrine from sympathetic nerve terminals, resulting in vasoconstriction, increased heart rate, enhanced myocardial contractility, and the activation of renin‐angiotensin‐aldosterone system, illustrating the intricate involvement of both the cardiovascular and renal systems (Esler, 2000). Recent non‐pharmacological treatment options for resistant hypertension include renal denervation and baroreceptor activation therapy.

Renal denervation involves the use of a catheter to deliver ultrasound or radiofrequency energy to ablate the sympathetic nerve fibers surrounding the renal arteries, thereby reducing excessive sympathetic activity (Azeez et al., 2024). On the other hand, baroreceptor activation therapy entails the electrical stimulation of the carotid sinus baroreceptors, which leads to a suppression of cardiovascular activity in the medullary centers, subsequently lowering peripheral sympathetic nerve activity and helping to control blood pressure (Biffi et al., 2024). However, the clinical selection of these invasive treatments remains a subject of ongoing debate.

Given these challenges, we focus on the neuromodulatory effects of magnetism to explore the potential of developing a new non‐invasive therapeutic approach. Interestingly, the effects of transcranial static magnetic field (tSMS) therapy on the brain have begun to be investigated in recent years, offering an alternative to transcranial magnetic stimulation (TMS). A study found that placing a 110–190 mT NdFeB magnet just above the primary motor cortex (M1) of the cerebral cortex for 10 min leads to a 25% reduction in motor evoked potentials (MEPs) (Oliviero et al., 2011), which has renewed the interest in SMF research. Furthermore, the inhibitory effects of tSMS last after the magnet is removed—lasting up to 30 min following 30 min of stimulation (Dileone et al., 2018; Oliviero et al., 2011; Silbert et al., 2013). Animal studies also have demonstrated that SMF decrease spontaneous neuronal activity and visual evoked potentials (Aguila et al., 2016). Another study investigated axon initiation segment (AIS) plasticity, finding that 6–48 h of SMF exposure altered the structure of the AIS in cortical neurons, particularly in inhibitory neurons, suggesting that SMF may influence neuronal excitability (Beros et al., 2024). While this study implies that SMF may affect voltage‐gated calcium channels, the exact mechanism remains unclear (Beros et al., 2024). More detailed studies indicate that generating a magnetic pressure gradient of about 10^−2^ Pa and a surface tension of 0.1–1 mN/m in brain tissue may enable SMF to activate mechanosensitive ion channels or mechanically stimulate voltage‐dependent channel function (Hernando et al., 2020). Moreover, Albuquerque et al., 2016 suggest that increased friction between ions and channels may alter ion conductance, but Hernando et al., 2020 reported that the Lorentz force exerted by the magnetic field on ions is unlikely to directly affect neuronal activity. Some researchers support the theory that moderate SMF in the range of 1–1000 mT can non‐invasively modulate neuronal activity through mechanisms such as the magnetization‐induced reorientation of phospholipid molecules (magnetic anisotropy), deformation (strain) of ion channels, or mechanical effects like rotation (Rosen, 2003; Hernando et al., 2020; Freire et al., 2020). Overall, while there is consensus that moderate SMF suppress neuronal excitability in the brain, no specific theory has emerged to explain the underlying mechanisms.

As previously mentioned, many studies have investigated the effects of SMF on the brain, providing valuable insights for medical applications owing to their low invasiveness and high usability. However, few studies have validated these findings using cells from the autonomic nervous system. Several studies employing patch‐clamp techniques have examined the relationship between SMF and ion channels in various cell types, such as primary sensory cells from the trigeminal ganglia (Lu et al., 2015) and cultured GH3 cells (Ad, 1996). These studies suggest an inhibitory effect of SMF on neuronal activity. We aim to explore the potential of therapeutic application of SMF technology for modulating the autonomic nervous system, particularly in conditions such as hypertension and arrhythmias associated with sympathetic overactivity. In this study, we investigate the effects of SMF on ion channels through electrophysiological and genetic analysis, using PC12 cells—a rat adrenal‐derived pheochromocytoma cell line that differentiates into sympathetic‐like cells.

## 2 MATERIALS AND METHODS

### 2.1 Cell culture and cell differentiation

Cultured PC12 cells (IFO50278) are obtained from the JCRB Cell Bank of the National Institutes of Biomedical Innovation and Nutrition. The cells are cultured and passaged (P12‐25) in RPMI medium supplemented with fetal bovine serum. The medium is replaced the day after seeding 7 × 10^4^ cells onto a 35‐mm dish, and NGF (Catalog No. CLMCNET‐001.1, Cedarlane Laboratories Ltd.) is added to a final concentration of 100 ng/mL. Medium replacement and NGF addition are performed every 3 days, and patch‐clamp measurements are conducted 6 ± 1 days after cell differentiation. Incubator conditions are maintained at 37°C and 5% CO₂ to ensure a suitable environment for cell culture.

### 2.2 Evaluation of differentiation by immunostaining

**FIGURE 1:**
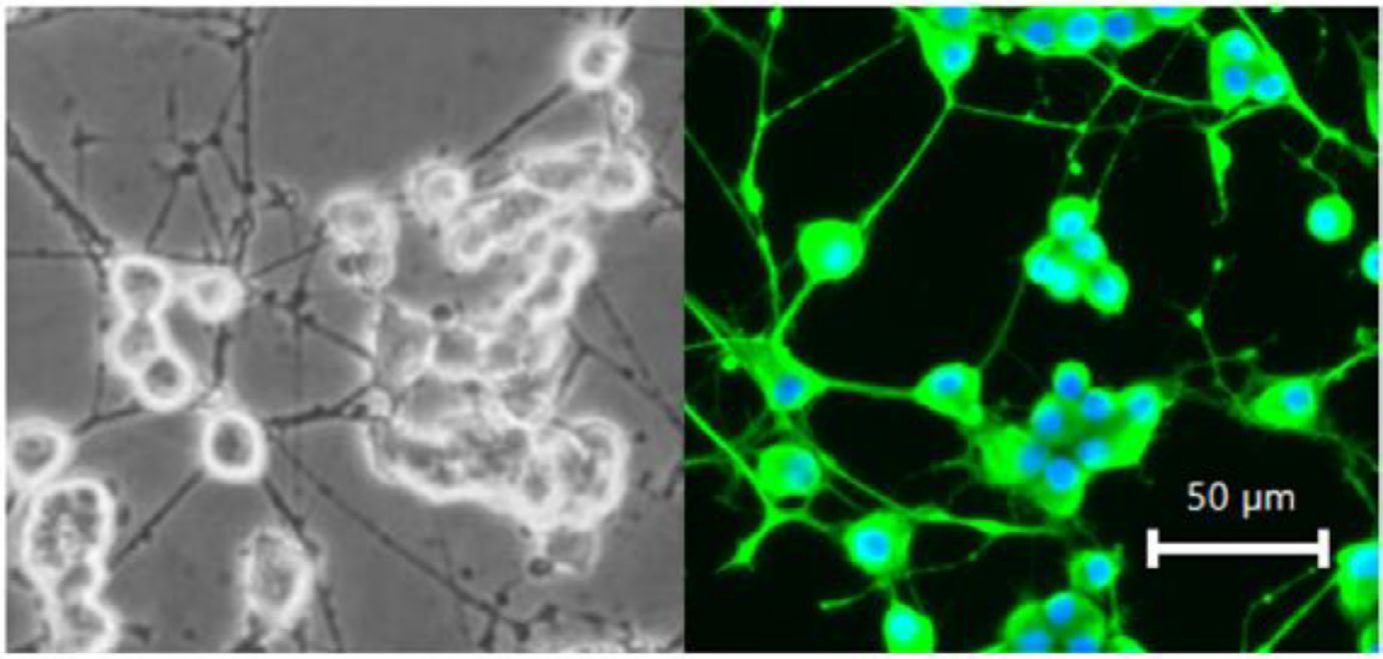
PC12 neurons. PC12 cells after the addition of NGF show axonal elongation and differentiation into sympathetic‐like neurons. Fluorescent staining labels tyrosine hydroxylase (TH), a marker for sympathetic neurons. Almost all cells have differentiated into sympathetic‐like cells on Day 6.

When NGF is added to PC12 cells, neuronal elongation can be observed after approximately 3 days. To confirm differentiation into sympathetic‐like cells, immunostaining for tyrosine hydroxylase (TH) is performed after NGF addition, following this procedure: First, PC12 cells are fixed in a 4% paraformaldehyde solution for 15 min at room temperature. Next, the cells are permeabilized with 0.1% Triton X‐100 (1:1000 dilution) for 10 min and blocked with 2% BSA in PBS for 1 h. For co‐staining, the cells are incubated with a rabbit‐derived TH antibody (1:200, overnight; catalog No. 25859‐1‐AP, PROTEINTECH). The following day, the cells are washed and blocked with PBS containing 5% BSA for 1 h at room temperature, then incubated with a goat anti‐rabbit IgG (H + L) cross‐adsorbed secondary antibody (1:500, overnight at 4°C; Alexa Fluor [TM] 488, Catalog No. A11008, Invitrogen) and DAPI (1:200, 1 h at room temperature; Catalog No. 62248, Thermo Scientific). (Figure 1). Images are acquired using an inverted fluorescence phase contrast microscope (BZ‐X800L, KEYENCE) with a 20x objective lens. For DAPI visualization, a DAPI‐V filter set was used with excitation at 395/25 nm, emission at 460/50 nm, and a dichroic mirror at 400 nm. For Alexa Fluor 488 visualization, a GFP‐B filter set was used with excitation at 470/40 nm, emission at 535/50 nm, and a dichroic mirror at 495 nm. The overlay images are captured using BZ‐X800 Analyzer software.

### 2.3 Magnetic field exposure

**FIGURE 2:**
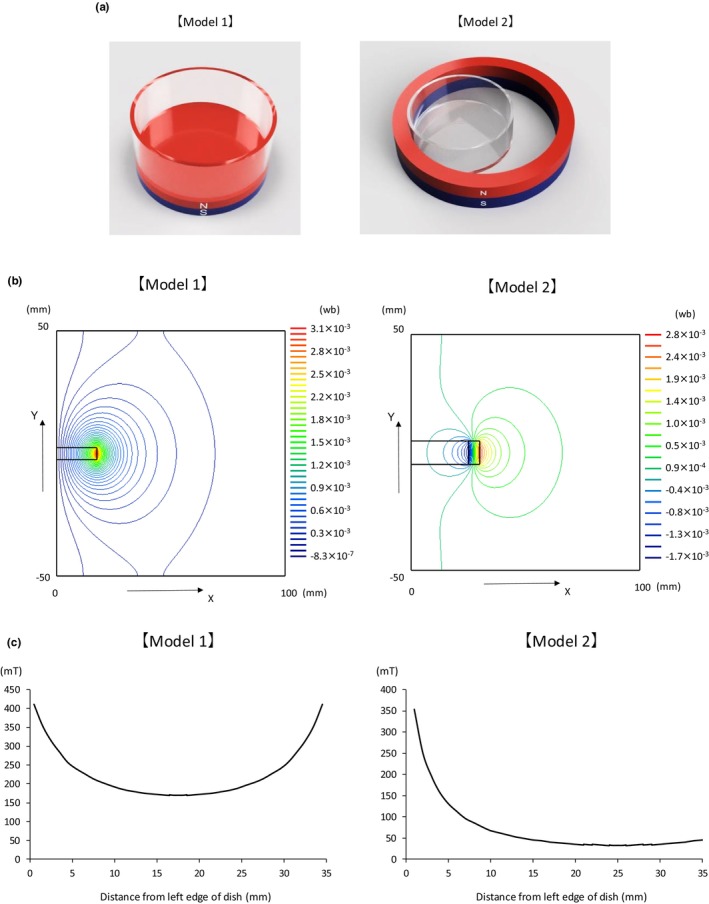
(a) Magnet. Model 1 is a disc‐shaped NdFeB magnet (φ35 × 5 mm), exposed by placing a 35 mm dish on top of the magnet. Model 2 is a ring‐shaped NdFeB magnet (φ60 × φ50 × 10 mm), exposed by positioning a dish along the left edge of the magnet. (b) Magnetic Flux Diagram. The distribution of the magnetic field is shown by the magnetic flux lines when the magnets of Model 1 and Model 2 are viewed from the side. The *x*‐axis and *y*‐axis represent the distance from the magnet (in mm), and the color scheme indicates the number of magnetic flux lines (in Weber, Wb). The magnetic flux density shown in (c) can be calculated by dividing the number of magnetic flux lines by the area. In this study, the thickness of the bottom of the 35 mm dish was assumed to be 1 mm, and magnetic field data corresponding to the positions of the cells on the dish were obtained through analysis. (c) Graph of magnetic flux density. For Model 1, cells on the dish are predicted to be exposed within the flux density range of 171–411 mT, while for Model 2, the range is 34–353 mT.

On each experimental day, two 35‐mm dishes, each containing 7 × 10^4^ PC12 cells, are prepared for the Sham and Mag groups. After 120–144 h of culture following the initial addition of NGF, only the Mag group is exposed to a disk magnet for the indicated period. A 35‐mm dish is placed on a disk‐shaped magnet with a diameter of 35 mm and a height of 5 mm (Neomag), as illustrated in Model 1 (Figure 2a). Magnetic field exposure times are set to 1, 6, or 18 h, depending on each experiment (Experiment 1–6). There was no obvious difference in cell morphology between the Mag group cells, which were magnetically exposed for 18 h, and the Sham group cells, which were cultured for 18 h without magnetic exposure. We also compared the membrane capacitance which reflects the cell size, and there was no significance between them (Experiment 3: 40.27 ± 16.84 pF and 53.29 ± 26.34 pF for Sham and Mag groups, respectively/Experiment 4: 56.44 ± 27.00 pF and 53.54 ± 23.70 pF for Sham and Mag groups, respectively). The predicted magnetic flux density at the bottom of the dish ranges from 171 to 411 mT (Figure 2b,c). The image of Model 2 depicts the arrangement of the magnets during the patch‐clamp procedure. For the Mag group, measurements are started immediately after removing the disk magnet and placing a ring‐shaped magnet (outer diameter 60 mm, inner diameter 50 mm, height 10 mm, Neomag) along the left edge of the dish, as shown in Model 2 (Figure 2a). The measurement duration for both the Sham and the Mag groups is 150 min following the replacement of the extracellular fluid in each dish.

In this configuration, the magnetic flux density at the bottom of the dish ranges from 34 to 353 mT (Figure 2b,c). Magnetic field analysis was performed using μ‐Excel (Mutec Corporation). A 3‐axis Gaussmeter (USB Handheld 3‐Axis Teslameter, SENIS), capable of measuring in the microtesla range, was used to measure the magnetic field. For background magnetic field measurements, the sensor was placed inside the incubator, and the values were recorded on an external computer with the incubator door closed. The maximum value was recorded every 15 s, with a total of 10 measurements, and calibration was performed after each measurement. Ambient magnetic fields were measured similarly, with the sensor placed in an area free of equipment or power sources within a 1‐m radius.

### 2.4 Whole‐cell patch‐clamp recordings

Patch pipettes are prepared using a SUTTER P‐97 puller, following a protocol designed to achieve a resistance of 3–8 MΩ by using the pipettes have an outer diameter of 1.5 mm and an inner diameter of 0.86 mm. The basal extracellular solution consists of the following (in mM): 145 NaCl, 5 KCl, 2 CaCl₂, 1 MgCl₂, 10 HEPES, and 10 D‐glucose monohydrate. The pH is adjusted to 7.3–7.5 using 10 N NaOH. After washing out the culture medium, the dish is filled with 2 mL of this extracellular solution. The intracellular solution for the patch pipettes contains (in mM): 135 KCl, 1 MgCl₂, 1 EGTA, 10 HEPES, and 20 D‐sucrose. Recordings are made and analyzed using pClamp 11 software (Molecular Devices). The total number of cells is shown in Table 1. The patch clamp experiments were conducted over three or more days in each experimental group, and they were combined. We also tried to avoid conducting experiments in the same order of groups each day. The I‐V curves were plotted by measuring the current amplitude at the end of each step pulse (495–500 ms after applying the voltage) for every current trace.

**TABLE 1:** Experimental designs for Experiments 1 through 6, including group settings and details on exposure period to the Model 1 magnet during cell culture, the type of recording solution used during patch clamp, and the presence, absence, or exposure period to the Model 2 magnet during patch clamp.

	Group settings	Model 1 exposure time during culture	Patch‐clamp recording solution	Model 2 exposure time during recordings
Experiment 1	Sham group (*n* = 17)	Sham: none	Normal	Sham: none
	Mag group (*n* = 19)	Mag: 1 h		Mag: up to 150 min
Experiment 2	Sham group (*n* = 22)	Sham: none	Normal	Sham: none
	Mag group (*n* = 18)	Mag: 6 h		Mag: up to 150 min
Experiment 3	Sham group (*n* = 21)	Sham: none	Normal	Sham: none
	Mag group (*n* = 20)	Mag: 18 h		Mag: up to 150 min
Experiment 4	Sham group (*n* = 28)	Sham: none	Normal	None
	Mag group (*n* = 24)	Mag: 18 h		
Experiment 5	Control group (*n* = 20)	18 h	Control: Normal	None
	4‐AP group (*n* = 20)		4‐AP: 1 mM	
	TEA group (*n* = 22)		TEA: 10 mM	
Experiment 6	Sham group (*n* = 28)	Sham: none	TEA: 10 mM	None
	Mag group (*n* = 27)	Mag: 18 h		

### 2.5 Kv channel inhibition experiments

Two different Kv channel inhibitors were used in Experiment 5. After exposing the cells to the magnetic field for 18 h, the medium was replaced with the basal extracellular solution in the control group. One group was treated with 4‐aminopyridine (4‐AP, Catalog No. A5006, Sigma‐Aldrich) dissolved in the extracellular solution to a final concentration of 1 mM, while another group was treated with tetraethylammonium (TEA, Catalog No. 90283, Sigma‐Aldrich) dissolved in the extracellular solution to a final concentration of 10 mM. In experiment 6, the medium was replaced with the same extracellular solution containing TEA as used in Experiment 5. Patch‐clamp measurements were then performed on the cells from the Sham and the Mag groups.

#### 2.5.1 The experimental design of this study is described below (Table 1)

##### Experiment 1

The Sham group receives no magnetic field exposure. The Mag group is exposed to the Model 1 magnet during culture for 1 h. Then, it is exposed to the Model 2 magnet during patch‐clamp recording (up to 150‐min). The culture medium was replaced with a recording solution just before patch‐clamp recording.

##### Experiment 2

The Sham group receives no magnetic field exposure. The Mag group is exposed to the Model 1 magnet during culture for 6 h. Then, it is exposed to the Model 2 magnet during patch‐clamp recording (up to 150‐min). The culture medium was replaced with a recording solution just before patch‐clamp recording.

##### Experiment 3

The Sham group receives no magnetic field exposure. The Mag group is exposed to the Model 1 magnet during culture for 18 h. Then, it is exposed to the Model 2 magnet during patch‐clamp recording (up to 150‐min). The culture medium was replaced with a recording solution just before patch‐clamp recording.

##### Experiment 4

The Sham group receives no magnetic field exposure. The Mag group is exposed to the Model 1 magnet during culture for 18 h, but there is no magnetic field exposure during the patch‐clamp recording. The culture medium was replaced with a recording solution just before patch‐clamp recording.

##### Experiment 5

All three groups are exposed to the Model 1 magnet for 18 h, but there is no magnetic field exposure during patch‐clamp recording (up to 150‐min). The culture medium was replaced with a recording solution just before patch‐clamp recording. The recording solution for the 4‐AP group contains 1 mM 4‐AP, while TEA group contains 10 mM TEA.

##### Experiment 6

The Sham group receives no magnetic field exposure. The Mag group is exposed to the Model 1 magnet for 18 h, but there is no magnetic field exposure during patch‐clamp recording (up to 150‐min). The culture medium was replaced with a recording solution with 10 mM TEA just before patch‐clamp recording.

### 2.6 RNA sequencing

PC12 cells are seeded at equal density in 35‐mm dishes, adopting the conditions used in patch‐clamp experiments. On day 5 of differentiation, dishes from both the Sham group (*n* = 3) and the Mag group (*n* = 3) are washed once with D‐PBS, then dissolved in QIAzol lysis Reagent (QIAGEN) and pipetted thoroughly, and immediately frozen at −80°C. RNA extraction and sequencing are performed in the Genome Analysis Laboratory at the Research Institute for Microbial Diseases, Osaka University. Data analysis is conducted using the iDEP 2.0 platform provided by South Dakota State University.

### 2.7 Statistical analysis

For the electrophysiological experiments, the student's *t*‐test is conducted, assuming equal variances. The normalized data are expressed as ±SD (standard error of the mean). The inactivating outward component, which includes the transient current, is defined as a decrease of 10% or more when comparing the maximum current value after the onset of voltage input with the current value near the end of the pulse. The chi‐squared test is performed to analyse the occurrence rate of this inactivating outward component.

## 3 RESULTS

### 3.1 Effects of SMF on PC12 neurons

**FIGURE 3:**
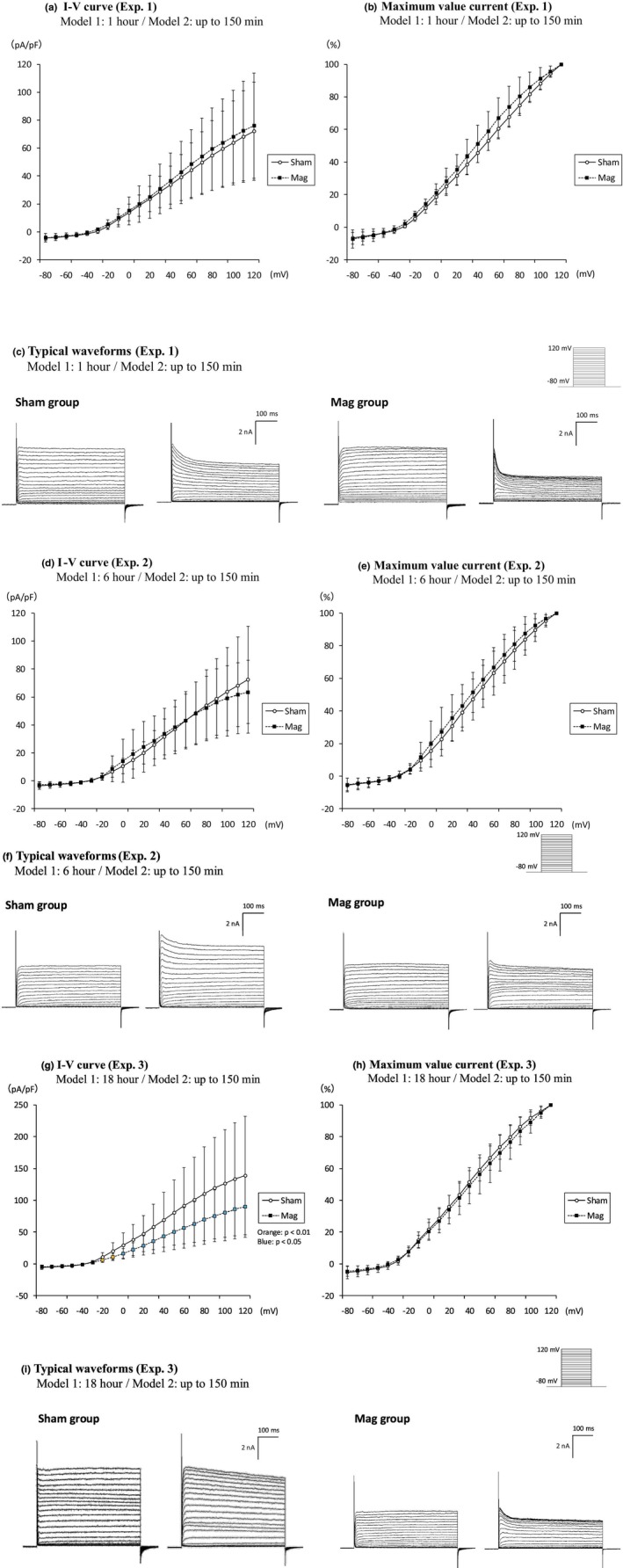
Current density for patch clamp measurements during the magnetic field exposure of Model 1 magnets for a specified time, followed by exposure of Model 2 for a specified time (Experiment 1–3). The number of n for each group represents the total number of independent cells measured. (a–c) Exposure time for Model 1: 1 h (Sham group *n* = 17, Mag group *n* = 19). (d–f) Exposure time for Model 1: 6 h (Sham group *n* = 22, Mag group *n* = 18). (g–i) Exposure time for Model 1: 18 h (Sham group *n* = 21, Mag group *n* = 20). Graphs showing the I–V curves and their ratio to the maximum value current, two typical waveforms of the Sham and Mag groups in sequence. The orange and light blue plots indicate significant differences at *p* < 0.01 and *p* < 0.05, respectively. The normalized data are expressed as ±SD. Statistical significance: Compared to the Sham group. The exposure period indicated on the label of each graph or waveform refers to the Mag group. No magnet exposure was conducted in the Sham group.

Whole‐cell patch‐clamp recordings were performed on PC12 neurons differentiated into sympathetic‐like cells for 6 ± 1 days. During the patch‐clamp recordings, step pulses were applied in 10 mV increments from −80 mV to +120 mV with the holding potential of −100 mV. The typical voltage‐dependent K^+^ current, observed as outward current, was mainly detected (e.g., Figure 3c left). On the other hand, the transient inward currents of voltage‐dependent Na^+^ channels were not detectable in the experiments. For the outward current, some population exhibited the voltage‐dependent inactivating components as shown in the right waveforms of both the Sham group and the Mag group in Figure 3c.

The Mag group was exposed to the Model 1 magnet for the specified exposure period, followed by the Model 2 magnet during the patch‐clamp recording. We examined the current density after 1 h of magnetic field exposure (Sham group: *n* = 17; Mag group: *n* = 19, Figure 3a–c)/Experiment 1), after 6 h of magnetic field exposure (Sham group: *n* = 22; Mag group: *n* = 18, Figure 3d–f)/Experiment 2), and after 18 h of magnetic field exposure (Sham group: *n* = 21; Mag group: *n* = 20, Figure 3g–i)/Experiment 3). When these results were compared using Student's *t*‐test, a significant difference from the Sham group was observed only in the experiment where cells were exposed to the magnet for 18 h. Throughout the experiments, there were no significant differences between groups in resting membrane potential (data not shown).

### 3.2 Persistence of SMF effects on PC12 neurons effects after magnet removal

**FIGURE 4:**
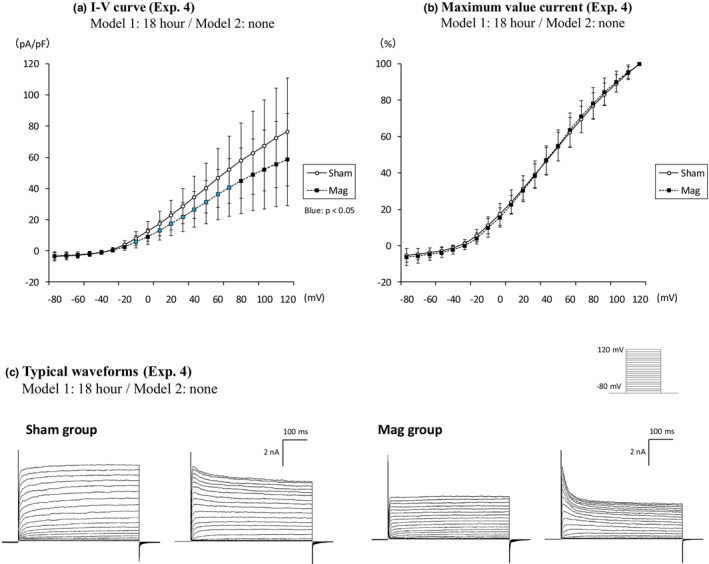
Current density for patch clamp measurements during the exposure of Model 1 magnets for a specified time, not followed by exposure of Model 2 for a specified time (Experiment 4). The number of n for each group represents the total number of independent cells measured. Exposure time for Model 1: 18 h (Sham group *n* = 28, Mag group *n* = 24). Graphs showing the I–V curves and their ratio to the maximum value current, two typical waveforms of the Sham and the Mag groups in sequence. The light blue plots indicate significant differences at *p* < 0.05, respectively. The normalized data are expressed as ±SD. Statistical significance: Compared to the Sham group. The exposure period indicated on the label of each graph or waveform refers to the Mag group. No magnet exposure was conducted in the Sham group.

In Experiment 4, cells in the Mag group were exposed to the Model 1 magnet for 18 h and then patch‐clamped without magnetic field exposure for 150 min during the measurement (Sham group: *n* = 28; Mag group: *n* = 24, Figure 4). A significant decrease in current density was observed in the Mag group compared to the Sham group, indicating that after a relatively long SMF exposure period of 18 h, the effects persisted for a certain period following the removal of the magnetic field.

### 3.3 SMF effects on Kv channel currents using inhibitors

**FIGURE 5:**
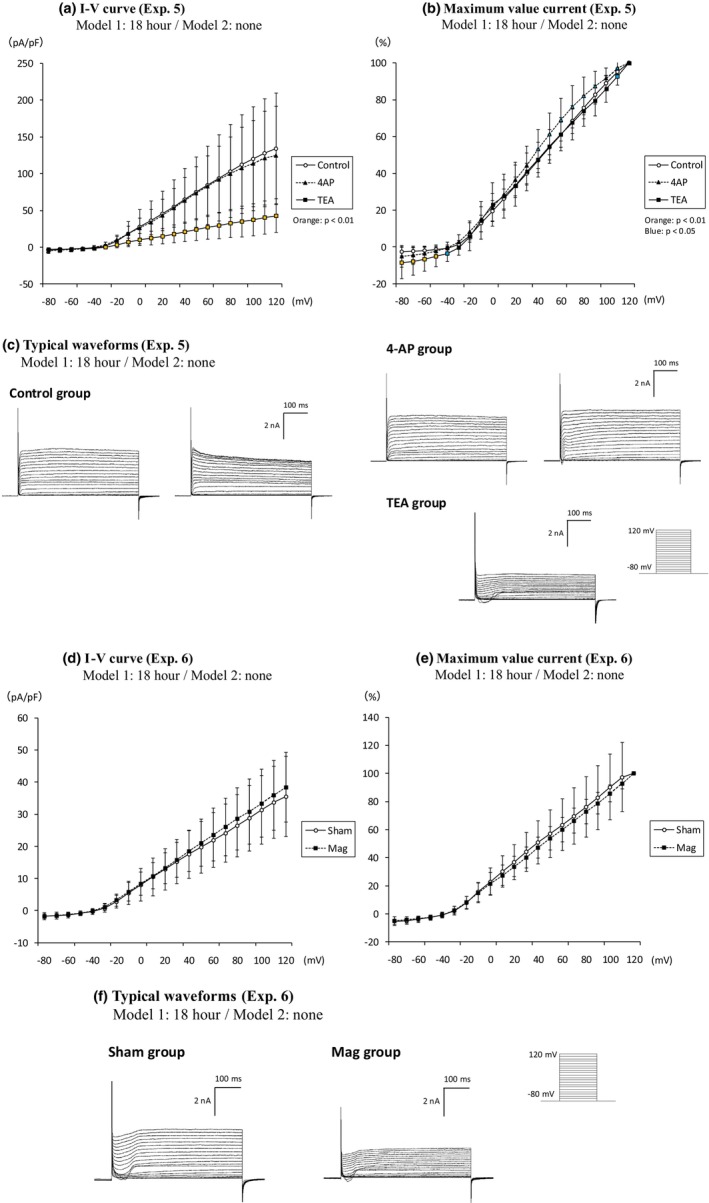
Experiments with Kv channel inhibitors (Experiment 5/6). The number of *n* for each group represents the total number of independent cells measured. (a–c) Experiment 5 show the selection of appropriate inhibitors for validation. Cells magnetically exposed with Model 1 for 18 h were patch clamped under three conditions: Control (*n* = 20, no Kv channel inhibitor), 4‐AP (*n* = 20, a Kv channel inhibitor), and TEA (*n* = 22, another Kv channel inhibitor). The current density was significantly lower with TEA. Model 2 was not exposed during patch clamping. (a) I–V curve, (b) their ratio to the maximum value current, (c) two typical waveforms in the control group, two typical waveforms in 4‐AP group and one typical waveform in TEA group. Light blue plots indicate significant differences at *p* < 0.05. (d–f) Experiment 6 illustrates patch clamping and current density under TEA without magnetic field exposure (*n* = 28) and with 18 h of magnetic field exposure (*n* = 27), showing no significant differences. (d) I–V curve, (e) their ratio to the maximum value current, (f) two typical waveform patterns in the Sham group and in the Mag group. The normalized data are expressed as ±SD. Statistical significance: Compared to the control/Sham group. The exposure period indicated on the label of each graph or waveform refers to the Mag group. No magnet exposure was conducted in the Sham group.

We hypothesized that inhibition of Kv channels by magnetic field exposure may account for the observed decrease in outward current density in the 18‐h magnet‐exposed group. We tested two well‐known Kv channel inhibitors, 4‐aminopyridine (4‐AP) and tetraethylammonium (TEA), expecting that the magnet field exposure effect on the outward currents will not be observed when the ionic currents are measured in the presence of the inhibitor. After 18 h of exposure to the magnet field, patch‐clamp recordings were performed by replacing the extracellular solution with one of three conditions: basal extracellular solution, basal extracellular solution with 1 mM 4‐AP, or basal extracellular solution with 10 mM TEA (control group: *n* = 20, 4‐AP group: *n* = 20, TEA group: *n* = 22; Experiment 5). Patch‐clamp recordings were conducted without magnetic field exposure for 150 min. The results showed a marked decrease in current density in the TEA group, while the 4‐AP group exhibited a slight, non‐significant decrease (Figure 5a). TEA treatment also exhibited the inward current that has not been observed in control, suggesting that the inward current was masked in the large Kv components in PC12. In Experiment 6, both the Sham group (*n* = 28) and the Mag group (*n* = 27), which were exposed to the magnet field for 18 h, had their medium replaced with extracellular solution with 10 mM TEA. Patch‐clamp recordings were then performed, without magnetic field exposure during the 150‐min recording. The difference in current density as observed in Experiment 4 was not observed under the TEA treatment in Experiment 6 (Figure 5d). We found that the previously observed difference in current density was not evident under TEA treatment (Figure 5d), indicating that the decrease of outward current via magnetic field exposure involves the voltage‐gated K^+^ channels.

### 3.4 Effects of SMF on the inactivating outward component of PC12 neurons

**FIGURE 6:**
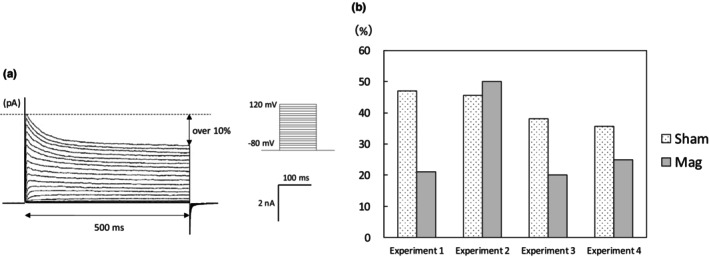
Estimation of inactivating outward component. (a) The inactivating outward component including the transient current is defined as a waveform that decreases by 10% or more when comparing the maximum current value after the start of the voltage input with the current value near the end of the pulse. (b) The occurrence rate of the inactivating outward component. The occurrence rate of this inactivating outward component was compared between the Sham and the Mag groups (Experiment 1–4). Bars indicate the percentage of occurrence in each group. The chi‐squared test was performed to analyze the occurrence rate of this inactivating outward component, but there was no significant difference in any group comparison.

We also examined the magnetic effect on the inactivating component of outward current. We defined the inactivating component as the transient current decrease of 10% or more when comparing the maximum current value after the onset of voltage input to the current value near the end of the pulse (Figure 6). We then examined the occurrence probability of this inactivating component both in the Sham and the Mag groups. A chi‐square test was used to analyze each group, revealing no significant difference in occurrence between the groups with and without magnetic field exposure (Table 2).

**TABLE 2:** Kv channel or other related genes. KCND3 (Kv4.3) is the only gene whose fold change in expression in the Sham and the Mag groups (*n* = 3 each) is more than 2.0‐fold and *p* < 0.01. References: Kcna: Kv1.x, Kcnb: Kv2.x, Kcnc: Kv3.x, Kcnd: Kv4.x, Kcnf: Kv5.x, Kcng: Kv6.x, Kcnq: Kv7.x, Kcnv: Kv8.x, Kcns: Kv9.x, Kcnh: Kv10.x, Kv11.x, Kv12.x., Kcnab: Voltage‐gated potassium channel beta subunit, Kcne: Voltage‐gated potassium channel subfamily, E.

Gene name	Description	NCBI gene ID [uid]	Fold change	Student's *t*‐test *p*‐value
Kcna1	Potassium voltage‐gated channel subfamily A member 1	24,520	1.000	
Kcna10	Potassium voltage‐gated channel subfamily A member 10	295,360	1.000	
Kcna2	Potassium voltage‐gated channel subfamily A member 2	25,468	1.000	
Kcna3	Potassium voltage‐gated channel subfamily A member 3	29,731	1.011	9.73E‐01
Kcna4	Potassium voltage‐gated channel subfamily A member 4	25,469	1.024	6.35E‐01
Kcna5	Potassium voltage‐gated channel subfamily A member 5	25,470	1.000	
Kcna6	Potassium voltage‐gated channel subfamily A member 6	64,358	1.000	
Kcna7	Potassium voltage‐gated channel subfamily A member 7	365,241	1.206	1.53E‐02
Kcnab1	Potassium voltage‐gated channel subfamily A member regulatory beta subunit 1	29,737	1.201	1.19E‐01
Kcnab2	Potassium voltage‐gated channel subfamily A regulatory beta subunit 2	29,738	1.045	7.26E‐01
Kcnab3	Potassium voltage‐gated channel subfamily A regulatory beta subunit 3	58,981	1.388	3.85E‐01
Kcnb1	Potassium voltage‐gated channel subfamily B member 1	25,736	1.017	8.63E‐01
Kcnb2	Potassium voltage‐gated channel subfamily B member 2	117,105	1.105	6.45E‐01
Kcnc1	Potassium voltage‐gated channel subfamily C member 1	25,327	−1.022	8.83E‐01
Kcnc2	Potassium voltage‐gated channel subfamily C member 2	246,153	1.000	
Kcnc3	Potassium voltage‐gated channel subfamily C member 3	117,101	−1.015	7.89E‐01
Kcnc4	Potassium voltage‐gated channel subfamily C member 4	684,516	−1.202	7.06E‐02
Kcnd1	Potassium voltage‐gated channel subfamily D member 1	116,695	1.047	2.38E‐01
Kcnd2	Potassium voltage‐gated channel subfamily D member 2	65,180	−1.273	3.68E‐01
Kcnd3	Potassium voltage‐gated channel subfamily D member 3	65,195	2.145	9.93E‐04
Kcne1	Potassium voltage‐gated channel subfamily E regulatory subunit 1	25,471	1.504	2.31E‐01
Kcne2	Potassium voltage‐gated channel subfamily E regulatory subunit 2	171,138	−1.033	3.80E‐01
Kcne3	Potassium voltage‐gated channel subfamily E regulatory subunit 3	63,883	1.181	3.74E‐01
Kcne4	Potassium voltage‐gated channel subfamily E regulatory subunit 4	367,302	−1.567	4.42E‐01
Kcne5	Potassium voltage‐gated channel subfamily E regulatory subunit 5	681,190	1.000	
Kcnf1	Potassium voltage‐gated channel modifier subfamily F member 1	298,908	1.000	
Kcng1	Potassium voltage‐gated channel modifier subfamily G member 1	296,395	−1.131	3.74E‐01
Kcng2	Potassium voltage‐gated channel modifier subfamily G member 2	307,234	1.238	1.70E‐01
Kcng3	Potassium voltage‐gated channel modifier subfamily G member 3	171,011	1.189	4.83E‐01
Kcng4	Potassium voltage‐gated channel modifier subfamily G member 4	307,900	1.000	
Kcnh1	Potassium voltage‐gated channel subfamily H member 1	65,198	1.062	5.05E‐01
Kcnh2	Potassium voltage‐gated channel subfamily H member 2	117,018	−1.057	2.08E‐01
Kcnh3	Potassium voltage‐gated channel subfamily H member 3	27,150	−1.307	2.76E‐01
Kcnh4	Potassium voltage‐gated channel subfamily H member 4	114,032	1.056	5.06E‐01
Kcnh5	Potassium voltage‐gated channel subfamily H member 5	171,146	1.000	
Kcnh6	Potassium voltage‐gated channel subfamily H member 6	116,745	1.141	3.98E‐02
Kcnh7	Potassium voltage‐gated channel subfamily H member 7	170,739	1.000	
Kcnh8	Potassium voltage‐gated channel subfamily H member 8	246,325	−1.021	9.10E‐01
Kcnip1	Potassium voltage‐gated channel interacting protein 1	65,023	1.000	
Kcnip2	Potassium voltage‐gated channel interacting protein 2	56,817	1.171	3.74E‐01
Kcnip3	Potassium voltage‐gated channel interacting protein 3	65,199	−1.408	1.28E‐02
Kcnip4	Potassium voltage‐gated channel interacting protein 4	259,243	1.000	
Kcnq1	Potassium voltage‐gated channel subfamily Q member 1	84,020	1.000	
Kcnq2	Potassium voltage‐gated channel subfamily Q member 2	170,848	1.039	8.10E‐01
Kcnq3	Potassium voltage‐gated channel subfamily Q member 3	29,682	1.086	7.91E‐01
Kcnq5	Potassium voltage‐gated channel subfamily Q member 5	259,273	−1.043	4.93E‐01
Kcns1	Potassium voltage‐gated channel, modifier subfamily S, member 1	117,023	1.000	
Kcns2	Potassium voltage‐gated channel, modifier subfamily S, member 2	66,022	1.000	
Kcns3	Potassium voltage‐gated channel, modifier subfamily S, member 3	83,588	1.000	
Kcnv1	Potassium voltage‐gated channel modifier subfamily V member 1	60,326	1.000	
Kcnv2	Potassium voltage‐gated channel modifier subfamily V member 2	294,065	1.000	
Kcnrg	Potassium channel regulator	305,947	1.028	9.25E‐01
Kcns1	Potassium voltage‐gated channel, modifier subfamily S, member 1	117,023	1.000	
Kcns2	Potassium voltage‐gated channel, modifier subfamily S, member 2	66,022	1.000	
Kcns3	Potassium voltage‐gated channel, modifier subfamily S, member 3	83,588	1.000	

### 3.5 Genetic analysis by RNA sequencing

**FIGURE 7:**
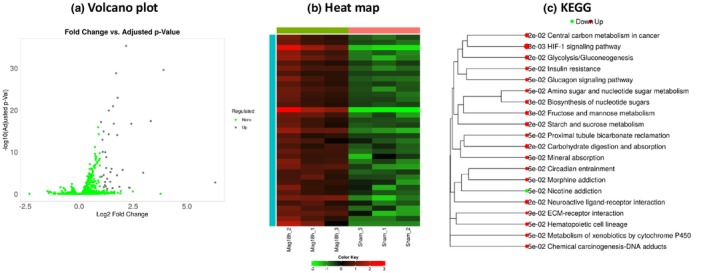
RNA sequencing analysis (a) Volcano plot. 37 genes were extracted by setting the fold change and FDR cutoff values. (b) Heat map. 37 genes were indicated in the sham group (*n* = 3) and the 18‐h magnet group (*n* = 3). (c) KEGG analysis was performed as part of the pathway analysis, showing 19 upregulated and 1 downregulated pathways.

Given the prolonged effect of 18‐h magnetic field exposure on current density, we conducted RNA sequencing analysis to investigate the effects of SMF on Kv channels or other related genes (Table 2). Comparisons were made between the Sham group (*n* = 3) and the Mag group (*n* = 3). This analysis identified 37 genes (Figure 7a) with expression level changes of more than 2‐fold, using a false discovery rate (FDR) cutoff of 0.1. A heatmap was generated to visualize the expression patterns of these genes (Figure 7b). KEGG analysis revealed 19 upregulated pathways and one downregulated pathway (Figure 7c). Notably, pathways related to neuroactive ligand‐receptor interactions—particularly the GABAB receptor pathway—and the circadian entrainment pathway were activated. These pathways included with several G proteins (Gi and Go), as well as pathways associated with Kir3 channel activation. Additionally, within the ECM‐receptor interaction pathway, there was a significant increase in laminin expression and a decrease in collagen expression.

## 4 DISCUSSION

Magnetic field exposure for a relatively long period (18 h) reduced the current density of Kv channels. Importantly, this effect persisted for some time after the removal of the magnet. The lack of a significant effect under TEA‐induced inhibition of Kv channels suggests that the SMF influences Kv channel function. However, except for Kv4.3, no differences in expression were observed in other Kv channels, indicating that SMF may affect Kv channels at the levels of protein translation or post‐translational modification.

The results of the KEGG analysis indicated that the GABAB pathway is more likely to be activated by magnetic field exposure, supported by a 1.9‐fold increase in the expression of KCTD16, an auxiliary subunit of the GABAB receptor (Zuo et al., 2019). Additionally, mRNA expression of RGS2, a signaling factor that enhances the activity of GABAB receptor‐binding Gproteins (Fowler, Aryal, Suen & Slesinger, 2007; Labouebe et al., 2007), increased 3.0‐fold. These changes may contribute to the inhibition of neuron excitability by promoting the opening of Kir3 channels.

### 4.1 Kv channels and current density reduction

The current density of PC12 neurons did not show significant differences after 1 or 6 h of magnetic field exposure, although a slight change began to emerge at the 6‐h mark. After 18 h, there was a clear decrease in current density, indicating a threshold between 6 and 18 h (Figure 3). In subsequent experiments (Figure 5), the difference in current density measured in the presence of TEA between the two groups substantially reduced when comparing conditions with and without magnetic field exposure (Figure 5d). This suggests that the magnetic field affects TEA‐sensitive Kv channels. Of note, we observed the transient inward current upon the addition of TEA, indicating that the current component was masked by the TEA‐sensitive K^+^ currents in the absence of the drug. This current component is likely due to voltage‐gated calcium channel activities which has been also previously reported in PC12 cells.

The occurrence rate of inactivation waveforms does not change regardless of the presence or absence of magnetic exposure (Figure 6), so we concluded that the magnetic effect in this study is not strongly related to the subtype of the inactivating voltage‐dependent potassium channels. In Figures 3g and 4a, where significant differences in current density were observed between the Sham and Mag groups, we analyzed the current at the end of the measurement duration (495–500 ms), primarily reflecting the non‐inactivating components. As an additional analysis, the inactivating components of the outward current were assessed by subtracting the current at the end (495–500 ms) from that at the start (3–18 ms) within the 500 ms measurement duration. No significant differences were observed between the two groups at any voltage input. These data suggest that SMF does not affect the I–V curves of inactivating component (Figure S1).

Unexpectedly, RNA sequencing results did not confirm an increase in gene expression for specific Kv channels except Kv4.3. While this result may not be consistent with our electrophysiological findings, it is still possible that magnetic field exposure affects processes such as the regulation of protein translation, post‐translational modifications, or the transport of proteins to the plasma membrane, which are necessary for the channel function. Alternatively, it is also possible that the expression or function of auxiliary subunits that regulate Kv channels have changed (Maffie, Dvoretskova, Bougis, Martin‐Eauclaire & Ruby, 2013).

Here we discuss the candidates of ion channels that are potentially sensitive to SMF in PC12 by the following criteria. (1) It is well expressed in PC12 neurons (FPKM>10). (2) It is well inhibited by TEA at concentrations below 20 mM. (3) It has slow or no inactivation property upon prolonged depolarization. Based on these criteria, Kv3.4, Kv7.2–7.3, and Kv10.1 could be listed as primary candidates contributing to SMF‐sensitive components (Blaine & Ribera, 2001; Dierich et al., 2018; Dixon & McKinnon, 1996; Gutman et al., 2003; Greene & Hoshi, 2017; Zemel, Ritter, Covarrubias & Muqeem, 2018). Notably, Kv7.2–7.3, which is important for regulating neuronal excitability and well expressed in PC12 neurons, has been shown to reduce M current by approximately 57% when blocked with 20 mM TEA (Nigro et al., 2014).

We also examined the expression of auxiliary subunits of Kv channels in PC12 cells. Kvβ2 (Kcnab), an auxiliary subunit of Kv3 family, was found to be expressed in PC12, and voltage‐gated potassium ion channel subfamily E member 2 (Kcne2), an auxiliary subunit of the Kv7 and Kv10 family, was fully expressed. However, these auxiliary subunits did not differ in their gene expression levels between the Sham and the Mag groups, and it would be also important topic whether the function of auxiliary subunits can be controlled by magnetic field.

### 4.2 Persistent SMF effect

Experiment 4 demonstrated that once PC12 neurons were exposed to the magnetic field for 18 h, the reduction in current density persisted for several hours after the magnet was removed.

Based on studies of static magnetic effects on the central nervous system, it has been shown that a relatively short 10‐min tSMS treatment reduces motor cortex excitability for 6 min (Oliviero et al., 2011) and that the amplitude of motor evoked potentials decrease for at least 30 min following a 30‐min stimulation (Dileone et al., 2018; Oliviero et al., 2011; Silbert et al., 2013). On the other hand, our findings suggest that the effect of longer exposure to magnetic fields persist even after the magnet is removed. This effect is probably mediated by the regulation of protein synthesis or gene expression of some Kv channels. Although we did not examine the recovery period after magnet removal in the present study, these regulation are generally transient, and it would probably recover to the basal level in a few days. Further research is needed to better define the relationship between magnetic field parameters, exposure period, and the time required for effects to subside in sympathetic neurons.

### 4.3 SMF effect on gene expression in PC12

Interestingly, we observed that SMF induces gene expression changes in a wide variety of molecules, and some of them largely contribute to the neuronal function.

For example, it is known that GABAB receptor and Kir3 channels are regulated through their interaction (David et al., 2006). Based on mRNA expression levels and KEGG analysis from RNA sequencing data, we predict that the activation pathway of GABAB receptors associated with Kir3 channels is likely to be promoted in cells exposed to magnetic fields in our study. We found that four genes related with the channels were upregulated more than 1.5‐fold in the 18‐h magnet‐exposed group compared to the Sham group: voltage‐gated potassium ion channel subfamily D member 3 (KCND3, Kv4.3) was upregulated 2.1‐fold (*p* < 0.01), potassium channel tetramerization domain containing 16 (KCTD16) increased 1.9‐fold (*p* < 0.05), chloride voltage‐gated channel Kb (CLC‐K2, Kb) increased 1.9‐fold (*p* < 0.01), and transient receptor potential cation channel subfamily C member 3 (TRPC3; Zhou, Matta & Zhou, 2008) also showed significant changes with a 1.6‐fold increase (*p* < 0.01).

We also observe the 2.3‐fold increase (*p* < 0.01) of mRNA expression in Synaptotagmin 13 (SYT13) in the Mag group. SYT13 is a calcium‐independent membrane protein primarily involved in the exocytosis of synaptic vesicles, which may help regulate synaptic activity and prevent excessive enhancement. It is also noteworthy that calcium/calmodulin‐dependent protein kinase II (CaMKII), which phosphorylates and regulates voltage‐dependent potassium ion channels, was reduced by 2.2‐fold (*p* < 0.01) in the Mag group. CaMKII also plays a role in synaptic plasticity and neurotransmitter release. Therefore, it is possible that a decrease in CaMKII contributes to the reduced function of voltage‐dependent potassium ion channels, leading to insufficient neurotransmission.

These results indicate that SMF could affect not only neuronal excitability but also synaptic function in sympathetic neurons.

### 4.4 The mechanisms of SMF action on neurons

The mechanisms by which SMF affect neurons are being investigated using a comprehensive range of research methods. We have reviewed various studies on how weak to moderate SMF affects neurons, including those discussing changes in plasma membrane potential, biomechanics, ion channel regulation, metabolism, and cell–cell interactions (ex. Fang et al., 2024; Liang et al., 2024; Nakamura, Nakao & Wakabayashi, 2019; Nikolic et al., 2013; Park et al., 2022; Saletnik et al., 2024). Specific studies, in addition to those mentioned in the Introduction, include the spatial rotation of anisotropic anti‐magnetic particles and the magnetophoretic pressure on cell membranes, both of which could influence cellular function (Hashemi & Abdolali, 2017). Also, Sinha et al., 2024 indicates that SMF exposure enhances the activity of chloride ion channels associated with the SLC26A11 transporter protein while suppressing action potential firing. As a new approach, the method of verifying the mechanism of static magnetic fields through the application of optogenetics and other techniques (Hernández‐Morales et al., 2024) is particularly interesting. Another noteworthy point is that we found a 2.5‐fold reduction in the expression of the interacting lysosomal protein (RILP), suggesting that cell migration may also be affected. Considering it from another viewpoint, although it is difficult to verify this in biological systems, it is believed that the magnetic field and radical pair mechanisms are closely related. Some reports have suggested that even small changes in the magnetic fields could affect chemical reactions by altering the quantum mechanical wave functions of colliding particle (Khurana et al., 2024; Player et al., 2021; Talbi et al., 2024).

Another important factor to consider regarding the experimental condition in the present study is the background magnetic field. It should be noted that even weak magnetic fields can lead to factors such as inhibited cancer cell proliferation (Raylman et al., 1996; Sun et al., 2023) and increased intracellular reactive oxygen species (Akimoto et al., 2024; Dobosz et al., 2024). The background magnetic field inside the incubator was measured to be 178 ± 8 μT, which is lower than the external magnetic field of 247 ± 15 μT and is not expected to significantly affect the magnetic field distribution or cell proliferation in the experimental setup. (Values are expressed as the mean ± SD of 10 measurements.) In this study, the Sham and Mag groups were cultured at a sufficient distance from each other in the same incubator.

## 5 CONCLUSION

In summary, this study found that exposure to moderate static magnetic fields (SMF) reduced the current density in sympathetic‐like PC12 neurons, indicating the effects on ion channels, particularly Kv channels. Notably, the effects of the magnetic field did not diminish immediately after the cessation of exposure; rather, they persisted for a certain period. The mechanism of moderate SMF exposure may involve molecular conformational changes in Kv channels and genetic modulation of pathways that inhibit neuronal excitation. We believe that the present study serves as a stepping stone for future research, emphasizing the need to investigate the effects of magnetic fields on peripheral sympathetic nerves. Unlike previous reports, this study is novel in that it was conducted in an experimental system that does not require a complicated magnet fixation setup. We hope that this work will encourage more active research in this field by utilizing inexpensive, readily available, commercially available magnets that fit into standard culture goods.

## AUTHOR CONTRIBUTIONS

EK, TK, and YO primarily conceptualized and designed the study. EK and TK conducted the electrophysiological experiments and analyzed the data. YO and SM supervised the overall study and were responsible for its execution. All authors contributed to editing and revising the manuscript and approved the final version.

## FUNDING INFORMATION

The Watanabe Foundation, Grant/Award Number: 290306. The Japan Association for the Advancement of Medical Equipment, Grant name: The 2024 Medical Technology Research and Development Grant.

## CONFLICT OF INTEREST STATEMENT

The authors declare no conflicts of interest.

## ETHICS STATEMENT

This study was conducted in accordance with ethical principles and guidelines for the use of cell cultures and laboratory practices. All cell lines used in this study were obtained from authorized sources, and no primary human or animal tissues were involved. The study adhered to institutional and national regulations regarding the handling of cell cultures, ensuring that all procedures followed established safety and ethical protocols.

## Supporting information


Figure S1.

## Data Availability

The data that support the findings of this study are available from the corresponding author upon reasonable request.

## References

[phy270236-bib-0001] Ad, R. (1996). Inhibition of calcium channel activation in GH3 cells by static magnetic fields. Biochimica et Biophysica Acta, 1282(1), 149–155. 10.1016/0005-2736(96)00053-3 8679652

[phy270236-bib-0002] Aguila, J. , Cudeiro, J. , & Rivadulla, C. (2016). Effects of static magnetic fields on the visual cortex: Reversible visual deficits and reduction of neuronal activity. Cerebral Cortex, 26(2), 628–638. 10.1093/cercor/bhu228 25260705

[phy270236-bib-0003] Akimoto, T. , Islam, M. R. , Nagasako, A. , Kishi, K. , Nakakaji, R. , Ohtake, M. , Hasumi, H. , Yamaguchi, T. , Yamada, S. , Yamamoto, T. , Ishikawa, Y. , & Umemura, M. (2024). Alternative magnetic field exposure suppresses tumor growth via metabolic reprogramming. Cancer Science, 115(8), 2686–2700. 10.1111/cas.16243 38877783 PMC11309929

[phy270236-bib-0004] Albuquerque, W. W. C. , Costa, R. M. P. B. , Fernandes, T. d. S. E. , & Porto, A. L. F. (2016). Evidences of the static magnetic field influence on cellular systems. Progress in Biophysics and Molecular Biology, 121(1), 16–28. 10.1016/j.pbiomolbio.2016.03.003 26975790

[phy270236-bib-0005] Azeez, G. A. , Thirunagari, M. , Fatima, N. , Anand, A. , Palvia, A. R. , Kaur, A. , & Nassar, S. T. (2024). The efficacy of renal denervation in treating resistant hypertension: A systematic review. Cureus, 16, e67007. 10.7759/cureus.67007 39286705 PMC11403650

[phy270236-bib-0006] Beros, J. L. , King, E. S. , Clarke, D. , Jaeschke‐Angi, L. , Rodger, J. , & Tang, A. D. (2024). Static magnetic stimulation induces structural plasticity at the axon initial segment of inhibitory cortical neurons. Scientific Reports, 14(1), 1479. 10.1038/s41598-024-51845-739 38233493 PMC10794225

[phy270236-bib-0007] Biffi, A. , Quarti‐Trevano, F. , Vanoli, J. , Dell'Oro, R. , Corrao, G. , Mancia, G. , & Grassi, G. (2024). Effects of acute carotid baroreceptor stimulation on sympathetic nerve traffic in resistant and uncontrolled hypertension: A systematic review and meta‐analysis. Hypertension Research, 47(7), 1962–1969. 10.1038/s41440-024-01704-9 38760523

[phy270236-bib-0008] Blaine, J. T. , & Ribera, A. B. (2001). Kv2 channels form delayed‐rectifier potassium channels in situ. The Journal of Neuroscience, 21(5), 1473–1480. 10.1523/jneurosci.21-05-01473.2001 11222637 PMC6762936

[phy270236-bib-0009] David, M. , Richer, M. , Mamarbachi, A. M. , Villeneuve, L. R. , Dupré, D. J. , & Hebert, T. E. (2006). Interactions between GABA‐B1 receptors and Kir 3 inwardly rectifying potassium channels. Cellular Signalling, 18(12), 2172–2181. 10.1016/j.cellsig.2006.05.014 16809021

[phy270236-bib-0010] Dierich, M. , Evers, S. , Wilke, B. U. , & Leitner, M. G. (2018). Inverse modulation of neuronal Kv12.1 and Kv11.1 channels by 4‐aminopyridine and NS1643. Frontiers in Molecular Neuroscience, 11, 11. 10.3389/fnmol.2018.00011 29440988 PMC5797642

[phy270236-bib-0011] Dileone, M. , Mordillo‐Mateos, L. , Oliviero, A. , & Foffani, G. (2018). Long‐lasting effects of transcranial static magnetic field stimulation on motor cortex excitability. Brain Stimulation, 11(4), 676–688. 10.1016/j.brs.2018.02.005 29500043

[phy270236-bib-0012] Dixon, J. E. , & McKinnon, D. (1996). Potassium Channel mRNA expression in prevertebral and paravertebral sympathetic neurons. European Journal of Neuroscience, 8(1), 183–191. 10.1111/j.1460-9568.1996.tb01179.x 8713462

[phy270236-bib-0013] Dobosz, B. , Gunia, E. , Kotarska, K. , Schroeder, G. , & Kurczewska, J. (2024). The effect of a magnetic field on the transport of functionalized magnetite nanoparticles into yeast cells. Applied Sciences, 14(4), 1343. 10.3390/app14041343

[phy270236-bib-0014] Esler, M. (2000). The sympathetic system and hypertension. American Journal of Hypertension, 13(6), S99–S105. 10.1016/S0895-7061(00)00225-9 10921528

[phy270236-bib-0015] Fang, F. , Liu, C. , Huang, Q. , Dong, C. , Zhang, G. , Jiang, J. , & Lu, S. (2024). Effect of static magnetic field on gene expression of human umbilical cord mesenchymal stem cells by transcriptome analysis. Sciences, 69(2), 281–288. 10.1016/j.advms.2024.06.001 38844059

[phy270236-bib-0016] Fowler, C. E. , Aryal, P. , Suen, K. F. , & Slesinger, P. A. (2007). Evidence for the40 association of GABA(B) receptors with Kir3 channel channels and regulators of G protein signalling (RGS4) proteins. The Journal of Physiology, 580(Pt 1), 51–65. 10.1113/jphysiol.2006.123216 17185339 PMC2075413

[phy270236-bib-0017] Freire, M. J. , Bernal‐Méndez, J. , & Pérez, A. T. (2020). The Lorentz force on ions in membrane channels of neurons as a mechanism for transcranial static magnetic stimulation. Electromagnetic Biology and Medicine, 39(4), 310–315. 10.1080/15368378.2020.1793172 32666841

[phy270236-bib-0018] Grassi, G. , Bombelli, M. , Buzzi, S. , Volpe, M. , & Brambilla, G. (2014). Neuroadrenergic disarray in pseudo‐resistant and resistant hypertension. Hypertension Research, 37(6), 479–483. 10.1038/hr.2014.25 24572914

[phy270236-bib-0019] Greene, D. L. , & Hoshi, N. (2017). Modulation of Kv7 channels and excitability in the brain. Cellular and Molecular Life Sciences, 74(3), 495–508. 10.1007/s00018-016-2359-y 27645822 PMC5243414

[phy270236-bib-0020] Gutman, G. A. , Chandy, K. G. , Adelman, J. P. , Aiyar, J. , Bayliss, D. A. , Clapham, D. E. , Covarriubias, M. , Desir, G. V. , Furuichi, K. , Ganetzky, B. , Garcia, M. L. , Grissmer, S. , Jan, L. Y. , Karschin, A. , Kim, D. , Kuperschmidt, S. , Kurachi, Y. , Lazdunski, M. , Lesage, F. , & Wymore, R. S. (2003). International Union of Pharmacology. XLI. Compendium of voltage‐gated ion channels: Potassium channels. Pharmacological Reviews, 55(4), 583–586. 10.1124/pr.55.4.9 14657415

[phy270236-bib-0021] Hashemi, S. , & Abdolali, A. (2017). Three‐dimensional analysis, modeling, and simulation of the effect of static magnetic fields on neurons. Bioelectromagnetics, 38(2), 128–136. 10.1002/bem.22019 27862074

[phy270236-bib-0022] Hernández‐Morales, M. , Morales‐Weil, K. , Han, S. M. , Han, V. , Tran, T. , Benner, E. J. , Pegram, K. , Meanor, J. , Miller, E. W. , Kramer, R. H. , & Liu, C. (2024). Electrophysiological mechanisms and validation of ferritin‐based Magnetogenetics for remote control of neurons. Journal of Neuroscience, 44(30), e1717232024. 10.1523/JNEUROSCI.1717-23.2024 38777598 PMC11270515

[phy270236-bib-0023] Hernando, A. , Galvez, F. , García, M. A. , Soto‐León, V. , Alonso‐Bonilla, C. , Aguilar, J. , & Oliviero, A. (2020). Effects of moderate static magnetic field on neural systems is a non‐invasive mechanical stimulation of the brain possible theoretically? Frontiers in Neuroscience, 14, 419. 10.3389/fnins.2020.0041941 32508563 PMC7248270

[phy270236-bib-0024] Khurana, D. , Jensen, R. H. , Giri, R. , Bocquel, J. , Andersen, U. L. , Berg‐Sørensen, K. , & Huck, A. (2024). Sensing of magnetic field effects in radical‐pair reactions using a quantum sensor. Physical Review Research, 6(1), 013218. 10.1103/PhysRevResearch.6.013218

[phy270236-bib-0025] Labouèbe, G. , Lomazzi, M. , Cruz, H. G. , Creton, C. , Luján, R. , Li, M. , Yanagawa, Y. , Obata, K. , Watanabe, M. , Wickman, K. , Boyer, S. B. , Slesinger, P. A. , & Lüscher, C. (2007). RGS2 modulates coupling between GABAB receptors and GIRK channels in dopamine neurons of the ventral tegmental area. Nature Neuroscience, 10(12), 1559–1568. 10.1038/nn2006 17965710

[phy270236-bib-0026] Liang, Q. , Chi, G. , Cirqueira, L. , Zhi, L. , Marasco, A. , Pilati, N. , Gunthorpe, M. J. , Alvaro, G. , Large, C. H. , Sauer, D. B. , Treptow, W. , & Covarrubias, M. (2024). The binding and mechanism of a positive allosteric modulator of Kv3 channels. Nature Communications, 15(1), 2533. 10.1038/s41467-024-46813-8 PMC10957983 38514618

[phy270236-bib-0027] Lu, X.‐W. , Du, L. , Kou, L. , Song, N. , Zhang, Y.‐J. , Wu, M.‐K. , & Shen, J.‐F. (2015). Effects of moderate static magnetic fields on the voltage‐gated sodium and calcium channel currents in trigeminal ganglion neurons. Biology and Medicine, 34(4), 285–292. 10.3109/15368378.2014.906448 24712748

[phy270236-bib-0028] Maffie, J. K. , Dvoretskova, E. , Bougis, P. E. , Martin‐Eauclaire, M.‐F. , & Rudy, B. (2013). Dipeptidyl‐peptidase‐like‐proteins confer high sensitivity to the scorpion toxin AmmTX3 to Kv4‐mediated A‐type K+ channels. The Journal of Physiology, 591(10), 2419–2427. 10.1113/jphysiol.2012.248831 23440961 PMC3678034

[phy270236-bib-0029] Nakamura, T. Y. , Nakao, S. , & Wakabayashi, S. (2019). Emerging roles of neuronal Ca2+ Sensor‐1 in cardiac and neuronal tissues: A mini review. Frontiers in Molecular42. Neuroscience, 12, 56. 10.3389/fnmol.2019.00056 PMC6409499 30886571

[phy270236-bib-0030] Nigro, M. J. , Mateos‐Aparicio, P. , & Storm, J. F. (2014). Expression and functional roles of Kv7/KCNQ/M‐channels in rat medial entorhinal cortex layer II stellate cells. The Journal of Neuroscience, 34(20), 6807–6812. 10.1523/JNEUROSCI.4153-13.2014 24828634 PMC6608108

[phy270236-bib-0031] Nikolić, L. , Bataveljić, D. , Andjus, P. R. , Nedeljković, M. , Todorović, D. , & Janać, B. (2013). Changes in the expression and current of the Na+/K+ pump in the snail nervous system after exposure to a static magnetic field. Biology, 216(Pt 18), 3531–3541. 10.1242/jeb.085332 23788713

[phy270236-bib-0032] Oliviero, A. , Mordillo‐Mateos, L. , Arias, P. , Panyavin, I. , Foffani, G. , & Aguilar, J. (2011). Transcranial static magnetic field stimulation of the human motor cortex. The Journal of Physiology, 589(Pt 20), 4949–4958. 10.1113/jphysiol.2011.211953 21807616 PMC3224885

[phy270236-bib-0034] Park, H.‐J. , Hong, H. , Thangam, R. , Song, M.‐G. , Kim, J.‐E. , Jo, E.‐H. , Jang, Y.‐J. , Choi, W.‐H. , Lee, M.‐Y. , Kang, H. , & Lee, K.‐B. (2022). Static and dynamic biomaterial engineering for cell modulation. Nanomaterials, 12(8), 1377. 10.3390/nano12081377 35458085 PMC9028203

[phy270236-bib-0035] Player, T. C. , Baxter, E. D. A. , Allatt, S. , & Hore, P. J. (2021). Amplification of weak magnetic field effects on oscillating reactions. Scientific Reports, 11(1), 9615. 10.1038/s41598-021-88871-8 33953230 PMC8100163

[phy270236-bib-0036] Raylman, R. R. , Clavo, A. C. , & Wahl, R. L. (1996). Exposure to strong static magnetic field slows the growth of human cancer cells in vitro. Bioelectromagnetics, 17(5), 358–363. 10.1002/(SICI)1521-186X(1996)17:5<358::AID-BEM2>3.0.CO;2-2 8915544

[phy270236-bib-0037] Rosen, A. D. (2003). Mechanism of action of moderate‐intensity static magnetic fields on biological systems. Cell Biochemistry and Biophysics, 39(2), 163–173. 10.1385/CBB:39:2:163 14515021

[phy270236-bib-0038] Saletnik, B. A. , Puchalska‐Sarna, A. , Saletnik, A. , Lipa, T. , Dobrzański, B. , & Puchalski, C. (2024). Static magnetic fields as a factor in modification of tissue and cell structure: A review. International Agrophysics, 38, 43–75. 10.31545/intagr/176998

[phy270236-bib-0040] Silbert, B. I. , Pevcic, D. D. , Patterson, H. I. , Windnagel, K. A. , & Thickbroom, G. W. (2013). Inverse correlation between resting motor threshold and corticomotor excitability after static magnetic stimulation of the human motor cortex. Brain Stimulation, 6(5), 817–820. 10.1016/j.brs.2013.03.007 23598254

[phy270236-bib-0041] Sinha, A. S. , Shibata, S. , Takamatsu, Y. , Akita, T. , Fukuda, A. , & Mima, T. (2024). Static magnetic field stimulation enhances shunting inhibition via a SLC26 family Cl−channel, inducing intrinsic plasticity. The Journal of Neuroscience, 44(9), e1324222024. 10.1523/JNEUROSCI.1324-22.2024 38302440 PMC10904086

[phy270236-bib-0042] Sun, J. , Tong, Y. , Jia, Y. , Jia, X. , Wang, H. , Chen, Y. , Wu, J. , Jin, W. , Ma, Z. , Cao, K. , Li, X. , Chen, Z. , & Yang, G. (2023). Effects of extremely low frequency electromagnetic fields on the tumor cell inhibition and the possible mechanism. Scientific Reports, 13(1), 6989. 10.1038/s41598-023-34144-5 37117238 PMC10147919

[phy270236-bib-0043] Talbi, O. , Zadeh‐Haghighi, H. , & Simon, C. (2024). The radical pair mechanism cannot explain telecommunication frequency effects on reactive oxygen species. 10.1101/2024.06.23.600261

[phy270236-bib-0044] Zemel, B. M. , Ritter, D. M. , Covarrubias, M. , & Muqeem, T. (2018). A‐type KV channels in dorsal root ganglion neurons: Diversity, function, and dysfunction. Frontiers in Molecular Neuroscience, 11, 253. 10.3389/fnmol.2018.00253 30127716 PMC6088260

[phy270236-bib-0045] Zhou, F.‐W. , Matta, S. G. , & Zhou, F.‐M. (2008). Constitutively active TRPC3 channels regulate basal ganglia output neurons. The Journal of Neuroscience: The Official Journal of the Society for Neuroscience, 28(2), 473–482. 10.1523/JNEUROSCI.3978-07.2008 18184790 PMC3652281

[phy270236-bib-0046] Zuo, H. , Glaaser, I. , Zhao, Y. , Kurinov, I. , Mosyak, L. , Wang, H. , Liu, J. , Park, J. , Frangaj, A. , Sturchler, E. , Zhou, M. , McDonald, P. , Geng, Y. , Slesinger, P. A. , & Fan, Q. R. (2019). Structural basis for auxiliary subunit KCTD16 regulation of the GABAB receptor. Proceedings of the National Academy of Sciences of the United States of America, 116(17), 8370–8379. 30971491 10.1073/pnas.1903024116 PMC6486783

