# Supplementary material for: Effects of moderate static magnetic fields on voltage‐gated potassium ion channels in sympathetic neuron‐like PC12 cells
Source: Physiol Rep. 2025 Mar 22;13(6):e70236. doi: 10.14814/phy2.70236 (PMC11928678; doi:10.14814/phy2.70236)
Supplement: Supplementary file 1 — Figure S1. [file PHY2-13-e70236-s001.zip › FIGURE S1.docx]

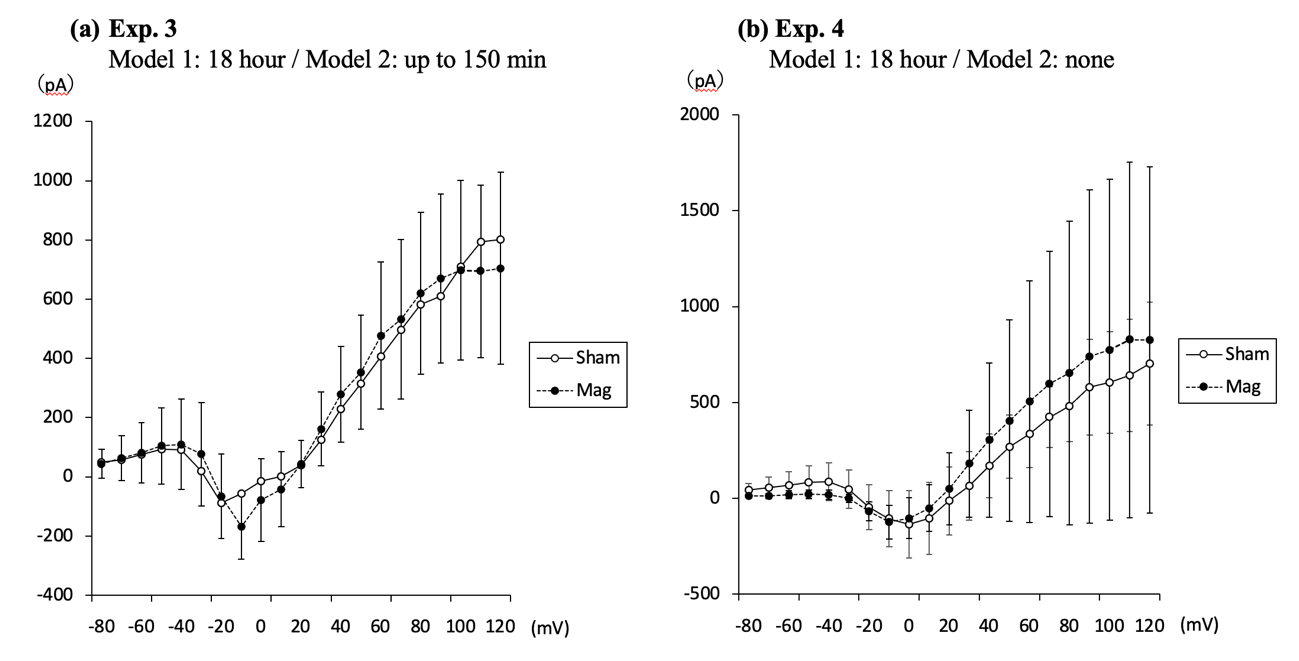


**FIGURE S1.** We investigated the inactivating component of the outward current by subtracting the current at the end (495-500 ms) from the start (0-20 ms) within the 500 ms measurement duration in Figures 3g and 4a. No significant difference was found between the two groups at any voltage input. There was no effect on the I-V curve regardless of the presence of the magnetic field. (a) Experiment 3 (Sham group n = 8, Mag group n = 4). (b) Experiment 4 (Sham group n = 10, Mag group n = 6). The normalized data are expressed as ± SD.
